# Arm circumference for age, arm circumference and weight-for-height z-score for the evaluation of severe acute malnutrition: a retrospective cohort study in eastern Democratic Republic of Congo

**DOI:** 10.1186/s12889-024-18083-y

**Published:** 2024-02-23

**Authors:** Gaylord Ngaboyeka, Ghislain Bisimwa, Anouk Neven, Pacifique Mwene-Batu, Richard Kambale, Emmanuel Ongezi, Christine Chimanuka, Joseph Ntagerwa, Serge Balolebwami, Francis Mulume, Oreste Battisti, Michèle Dramaix, Philippe Donnen

**Affiliations:** 1 Ecole Régionale de Santé Publique, Université Catholique de Bukavu, Bukavu, Democratic Republic of Congo; 2 Ecole de Santé Publique, Université Libre de Bruxelles, Brussels, Belgium; 3 Nutritional department, Centre de Recherche en Sciences Naturelles, Lwiro, Democratic Republic of Congo; 4 Competence Center for Methodology and Statistics, Luxembourg Institute of Health, Strassen, Luxembourg; 5 Hôpital Provincial General de Reference de Bukavu, Université Catholique de Bukavu, Bukavu, Democratic Republic of Congo; 6 Département de sciences cliniques, Faculté de médecine, Université de Liège, Liège, Belgique; 7 Faculté de Médecine, Université de Kaziba, Kaziba, Democratic Republic of Congo; 8 Institut Supérieur des Techniques Médicales Kanyamulande, Walungu, Democratic Republic of Congo

**Keywords:** Severe acute malnutrition, MUAC, WHZ, MUACZ, Concordance, DR Congo

## Abstract

**Background:**

Little is known about the use of mid-upper arm circumference for age (MUACZ) for diagnosing of severe acute malnutrition (SAM) and its correlation with WHZ (weight-for-height Z-score) in an area endemic for severe acute malnutrition (SAM) and with a high prevalence of kwashiorkor. Our study aims to analyze the concordance between the diagnostic criteria of SAM in a region presenting these characteristics.

**Methods:**

We analyzed a database of children admitted from 1987 to 2008 for the management of SAM in Eastern Democratic Republic of Congo. Anthropometric indicators (z-score) were calculated and classified into 3 categories according to WHO standards. Cohen’s kappa coefficient (κ) was calculated to assess the concordance between these indicators.

**Results:**

Out of the 9969 selected children aged 6 to 59 months, 30.2% had nutritional edema, 70.1% had a height-for-age (HAZ) z-score <-2, 11.5% WHZ<-3 z-score, 14.9% had a MUAC < 115 mm and 21.8% had a MUACZ <-3 z-score. With the classic combination WHZ and MUAC, 36% of children with SAM had both criteria at the same time and MUAC alone being the indicator that recruited more children with SAM (77%) compared with 65% with WHZ only. By replacing MUAC with MUACZ, 34% of SAM children fulfilled both criteria, WHZ and MUACZ. MUACZ alone recruited more children with SAM (88%) compared with 46% with WHZ alone. Considering these three indicators together, MUACZ remained the indicator that recruited more children with SAM (85%). WHZ and MUAC showed a moderate agreement [ κ (95% CI) = 0.408(0.392–0.424)], WHZ and MUACZ a weak agreement [ κ (95% CI) = 0.363(0.347–0.379)] and MUAC and MUACZ a good agreement [ κ (95% CI) = 0.604 (0.590–0.618)].

**Conclusion:**

Adjusting MUAC according to age improves its effectiveness in identifying severe acute malnutrition. With low concordance, MUAC and WHZ remain complementary in our context. MUACZ proves to be crucial, especially in the presence of kwashiorkor and chronic malnutrition, becoming a valuable tool for assessing severe acute malnutrition in our context.

**Supplementary Information:**

The online version contains supplementary material available at 10.1186/s12889-024-18083-y.

## Background

Acute malnutrition (AM) is a public health problem worldwide, predominating in low-income countries [1–4]. It significantly increases the risk of morbidity and mortality in children under 5 years and is responsible for 13% of the deaths among these children worldwide [5]. 60% of all deaths from AM are attributable to its severe form [5]. In the community, the risk of death is twelve times higher in children with severe acute malnutrition (SAM) compared to well-nourished children [6]. Studies show that the early identification and management of malnourished children contributes greatly to prevent the lethality of malnutrition [7].

Significant progress has been made in the detection and management of AM. More than 20 years ago, the treatment of SAM was carried out exclusively in hospital settings, an approach that has been both expensive and limited in term of coverage. Starting from a purely medical model, an approach known as “Community Management of Acute Malnutrition (CMAM)” was first introduced in 2001 with the aim of decentralizing therapeutic care in the community. In the last decade, this approach has been implemented in emergency and routine situations to prevent the mortality of children suffering from SAM. To date, there is a lot of data from various countries that demonstrates the success of this model [8–13].

To determine admission to SAM programs for children aged 6–59 months, WHO recommends at least one of the following three criteria: (i) a weight-for-height Z-score (WHZ) <-3 when compared to 2006 WHO growth standards; (ii) a Mid-Upper Arm circumference (MUAC) < 115 mm; and (iii) the presence of nutritional edema [14].

Although apart from the presence of edema, WHZ and MUAC aim to measure SAM, many studies have shown that these two indices do not identify the same children with SAM. A 2009 World Health Organization (WHO) report [15] noted that of all children identified as having AM determined by either indicator, only about 40% were identified by both. This diagnostic discrepancy has been observed in multiple studies [16–21]. By analyzing data from 14,409 children from 4 countries, Roberfroid et al. [21] found that only 28.5% of children defined as global acutely malnourished (GAM) were diagnosed with both low MUAC and low WHZ. Furthermore, Grellety et al. [19], after analyzing data from 1832 surveys containing measurements of 1,384,068 children from 47 countries, observed that only 28.2% of children defined as having GAM and 16.5% SAM were diagnosed by MUAC and WHZ. The proportion of children identified by both indicators in studies varied by country but was consistently below 40%. This poor agreement has also been reported in more recent studies [22–23]. The importance of concordance analysis lies in its ability to enhance the quality of nutritional assessments, guide programmatic choices, and contribute to the overall advancement of knowledge in the field [24]. By shedding light on the reliability of indicators, this analysis provides crucial insights into clinical relevance and effectiveness in specific contexts. Moreover, it steers programmatic decisions by highlighting the complementarity of indicators and uncovering nuances in child recruitment, thereby contributing to a more comprehensive and tailored approach.

Researchers have partly explained this discrepancy by the fact that MUAC does not take age and sex into account. As such, MUAC for age (MUACZ) might have greater diagnostic concordance with WHZ than with MUAC given that MUACZ and WHZ are both adjusted for age and sex [23, 25–27]. Indeed, the MUACZ indicator is calculated similarly to the WHZ, both of which compare a child’s anthropometric measurements to an international reference population to identify AM in children aged 6–59 months. However, preliminary results from Somalia do not support the hypothesis that WHZ and MUACZ have high diagnostic concordance. Custodio et al. [26] found that although estimates of acute malnutrition (AM) prevalence were similar for WHZ and MUACZ, they did not screen for the same children with AM. They found that the proportion of children with AM identified by the WHZ and MUACZ criteria (28.3%) was higher than the proportion of children identified by the conventional MUAC and WHZ criteria (18.1%).

On the other hand, the use of MUAC has been increasingly encouraged in humanitarian contexts due to the simplicity of its measurement and low cost. Indeed, screening for SAM using MUAC has been shown to be feasible by low-literacy community members with limited training. Some studies support the exclusive use of MUAC on the basis that it was a better predictor of mortality than WHZ [17, 28–30]. However, this was not observed in all studies and only a limited number of these studies assessed the relationship between these indicators and the risk of mortality in the community in the absence of management [31–35].

Not all of these studies compared mortality risk according to the different stages of definition of malnutrition for each outcome (normal, moderate and severe). Hossain et al. [25] demonstrated that the existing thresholds recommended for the identification of SAM may not effectively capture the majority of Bangladeshi children with SAM. They proposed adjustments in the form of revised thresholds, advocating for higher values particularly in the case of older children. According to them, the respective thresholds for MUAC to better recruit severe (WHZ <-3) and moderate (WHZ <-2) acute malnutrition would be < 120 and < 125 mm for children aged 6 to 24 months, < 125 and < 135 mm for children aged 25 to 36 months and < 135 and < 140 mm for children aged 37 to 60 months [25].

A better diagnostic concordance between MUACZ and WHZ would have practical implications, as screening children with MUACZ or WHZ indicators is more complex than MUAC [27, 36].

Despite discrepancies between MUAC and WHZ in the diagnosis of SAM and in their relationship with mortality, few studies have analyzed the different diagnostic SAM criteria based on hospital data with large sample sizes, such as those from community surveys. Studies carried out in a hospital environment often have the advantage of high data quality and diagnostic precision and allow studying the impact of confounding factors. However, it is worth noting that CMAM is generally implemented in the community. The presence of large hospital databases can be explained by the need for high-quality data to study these specific diagnostic criteria.

In the Democratic Republic of Congo (DRC), malnutrition still remains one of the major public health problems, with a prevalence of 41,8% of chronic malnutrition in 2018 [37]. In South Kivu, one of 26 provinces of the DRC (located in the east of the country), malnutrition has been endemic since the 1960s [38]. One out of two children under the age of 5 suffers from chronic malnutrition (CM) and 8% suffer from AM with the predominance of kwashiorkor [37, 39]. Some studies, using data from community-based surveys conducted in several countries including DRC demonstrate that MUAC identifies more children with SAM than WHZ, and that there are discrepancies between MUAC and WHZ in SAM diagnosis as well as between their association with mortality [19, 21–23].

Our study aims to evaluate the concordance between WHZ and MUAC on the one hand, and WHZ and MUACZ on the other hand from a database of children followed at Hôpital Pédiatrique de Lwiro (HPL) in DRC.

## Methods

### Study area, design and population

This was a retrospective cohort study of children admitted for SAM from 1987 to 2008 at the HPL of the Centre de Recherche en Sciences Naturelles de Lwiro (CRSN-Lwiro), Southern-Kivu Province, 50 km from the city of Bukavu, in the DRC. The CRSN was created in 1947 and is located in the health zones (HZ) of Katana and Miti-Murhesa in South Kivu [40]. The HZs of Miti-Murhesa and Katana are respectively located 33 and 40 km from the city of Bukavu (headquarters of the province of South Kivu). CRSN organizes its activities in four research departments, namely: biology, geophysics, nutrition, and documentation. The nutrition department includes a pediatric hospital and several integrated health centers monitoring the health and nutrition status of children in the community [40].

The HPL is one of the first structures that has been involved in the management of malnutrition in DRC. With a capacity of 70 beds, in the 1970s, HPL functioned as a nutritional rehabilitation center, admitting only children suffering from SAM. But since the 1980s, it has been considered a reference hospital for all pediatric pathologies. Research activities were also carried out there. It had between 5 and 7 doctors. Consultations were provided by a general practitioner under the supervision of a pediatrician [40, 41].

A team of researchers, supported by the Scientific and Medical Center of the Free University of Brussels for its Cooperation Activities (CEMUBAC), has developed a model aimed at facilitating the management of data and programs related to SAM since the 1980s. This model also includes the digitization of hospital data since 1986. It is important to note that this model does not replace national protocols for the medical management of SAM, but rather is designed in accordance with these protocols to ensure effective complementarity. The electronic file contained sociodemographic, anthropometric, clinical, and biological data collected from hospitalized patients from 1987 to 2008, from admission to discharge [40].

At the time, the developed model took into account both diagnostic criteria and treatment-related aspects for Severe Acute Malnutrition (SAM). Specifically, the diagnosis at HPL was based on specific criteria, such as the Weight-for-Height Z-score (WHZ), relative to a locally established growth curve by DeMaeyer in 1959, even though this curve was not published [41–43]. Regarding nutritional treatment, it evolved in three periods, all in compliance with national recommendations. The first period (1987–1994) involved the use of MASOSO porridge, the second period (1994–1996) introduced High-Energy Milk (HEM), while the third period (August 1996-December 2007) replaced HEM with therapeutic milk F-75 in the first phase of treatment and F-100 in the second phase [41–43].

For our study, the nutritional status of patients was reassessed upon admission based on the WHO 2006 growth standards, and a new classification was established according to these updated criteria. We also explained this in another article published on this cohort [40].

The subjects retained in the analysis were those aged 6 to 59 months and in whom the anthropometric parameters at admission, in particular weight, height, and arm circumference, the presence of nutritional edema, the infectious status and the exit status had been collected. Children with missing data for at least one of these variables have been excluded. We also excluded subjects with values outside the limits defined by the WHO for z-scores and anthropometric parameters [15].

**Fig. 1:**
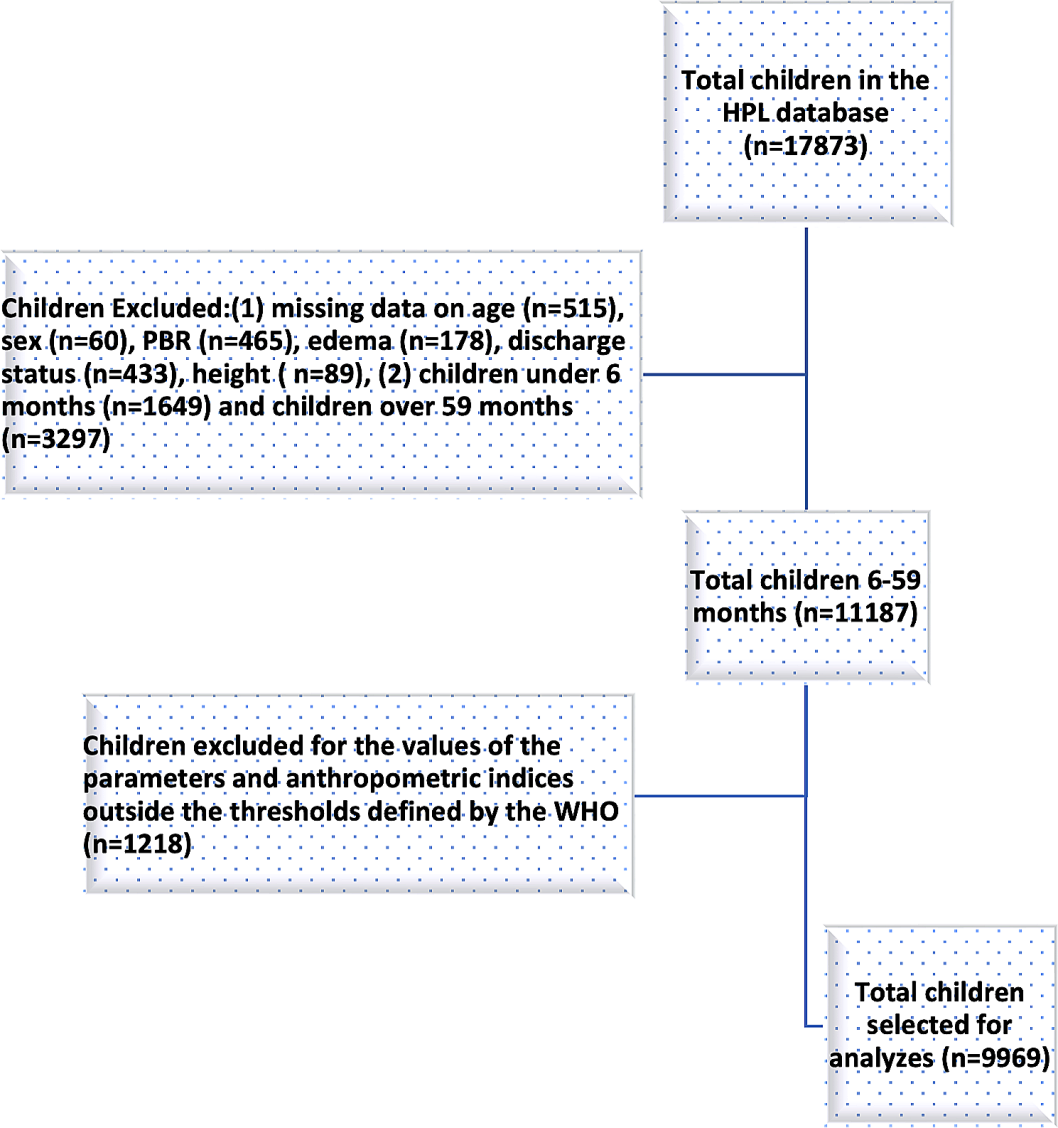
Flow chart of subjects selected for the study [44]

In the original HPL database from 1987 to 2008, a total of 17,873 children were recorded. Out these children, 11,187 were aged 6 to 59 months and had complete data for the study variables of interest. Of these, 1218 (10.9%) additional children were excluded because at least one of their anthropometric parameters exceeded WHO-defined thresholds, leaving 9969 children for our analyses [40] (Fig. 1).

It is important to note that this period predates the implementation of CMAM, and the children included in the study were treated at the hospital. Therefore, the records may not reflect the current reality of malnutrition management programs in the community.

### Variables

The variables used in the analysis were age, sex, weight, height, arm circumference, and the presence or absence of nutritional edema. We assessed nutritional status against WHO charts (40) and obtained z-scores for height-for-age (HAZ), mid-upper arm circumference-for-age (MUACZ), and weight-for-height (WHZ). The anthropometric indicators were each classified into 3 categories: [1] normal, [2] moderate acute malnutrition, and [3] severe acute malnutrition, in accordance with WHO standards (Table 1). It is important to note that the database used in our analysis includes individual measurements for each child, thereby enabling a specific assessment of anthropometric indicators such as MUAC, WHZ, and MUACZ for each participant.

**Table 1:** Definition of categories of MUAC, weight for height (WHZ), height for age (HAZ) and weight for age (WAZ)

	Acute malnutrition	Stature growth retardation	Underweight
Severe	At least one of the 3 criteria WHZ < -3 MUAC < 115 mm Edema	HAZ < -3	WAZ < 3
Moderate	At least one of the 2 criteria -3 ≤ WHZ < -2 115 ≤ MUAC < 125 mm No edema	-3 ≤ HAZ <-2	-3 ≤ WAZ <-2
Normal state	The 3 criteria WHZ ≥ -2 MUAC ≥ 125 mm No edema	HAZ ≥ -2	WAZ ≥ -2

MUAC: Mid-Upper arm circumference; WAZ: Weight-for-age Z-score; HAZ: height for age Z-score; WAZ: Weight for age Z-score

SAM was defined based on the latest WHO recommendation by the presence of nutritional edema and/or by MUAC < 115 mm and/or WHZ Z-score < -3. Apart from nutritional edema, we then classified SAM children by MUAC only, WHZ only and both MUAC and WHZ. Finally, chronic malnutrition (CM) was defined by a height-for-age (HAZ) Z-score < − 2 [15, 40].

To study MUACZ as diagnostic criteria, we inserted the latter into the SAM definition. It defines severe AM as MUCAZ < -3, moderate as a MUCAZ between − 3 and − 2 Z-score and normal state as a MUCAZ >- 2.

### Statistical analysis

The absolute and relative frequencies were used to describe categorical variables, encompassing both qualitative and ordinal variables. For the description of quantitative variables, the median with the interquartile range or the mean with the standard deviation were employed based on their distribution. Cohen’s kappa coefficient (κ) was calculated to assess the concordance between WHZ and MUAC on the one hand and WHZ and MUACZ on the other hand. We then assessed concordance after stratification by sex, age category, presence of edema, and CM. To qualify the degree of concordance, the values of κ were classified according to conventional thresholds κ ≤ 0.20, poor; 0.21 ≤ κ ≤ 0.40, low; 0.41 ≤ κ ≤ 0.60, moderate; 0.61 ≤ κ ≤ 0.80, good; and 0.81 ≤ κ ≤ 1.00, excellent [45].

## Results

### General characteristics of the studied population

Our results (Table 2) show that out of 9969 children, 30.2% had nutritional edema, 11.5% had a WHZ<-3, 14.9% a MUAC < 115 mm, 21.8% a MUACZ<-3 and 70.1% had chronic malnutrition.

**Table 2:** General characteristics of children aged 6 to 59 months hospitalized between 1987 and 2008 at the Pediatric Hospital of Lwiro (HPL), in eastern DRC

	*n* = 9969
	n (%) or Mean (SD) or Median (Min-Max)
Gender	
Boy	53.3
Girl	46.7
Age (months)	23 (6–59)
6 − 11	22.8
12 − 23	29.1
24 − 59	48.1
WHZ	-1.25 (1.39)
WHZ < − 3	11.5
−3 ≤ WHZ < − 2	16.9
−2 ≤ WHZ	71.6
HAZ	-2.84 (1.79)
HAZ < − 3	48.9
−3 ≤ HAZ < − 2	21.2
−2 ≤ HAZ	30.9
WAZ	-2.48 (1.5)
WAZ < − 3	39.1
−3 ≤ WAZ < − 2	23.4
−2 ≤ WAZ	37.5
MUAC (mm)	131.17 (16.55)
< 115	14.9
115 ≤ MUAC < 125	16.9
> 125	68.2
MUACZ	-1.81(1.55)
MUACZ < − 3	21.8
−3 ≤ MUACZ < − 2	20.4
−2 ≤ MUACZ	57.8
Edema	
No	69.8
Yes	30.2

Mean (SD); Median (Min-Max); WHZ: Weight-for-height Z - score; MUAC: mid upper arm circumference; MUACZ: mid upper arm circumference for age Z score; WAZ: Weight for age Z-score; HAZ: height for age Z-score. Z-score based on WHO curves [44]

### Concordance between WHZ and MUAC for the diagnosis of acute malnutrition in children aged 6 to 59 months

Our results showed that the concordance between WHZ and MUAC was moderate and that it remained virtually unchanged in the presence or absence of nutritional edema (Table 3).

**Table 3:** Concordance between WHZ and MUAC (mm) for the diagnosis of Acute Malnutrition in children aged 6 to 59 months admitted to HPL from 1987 to 2008, eastern DRC

	WHZ < − 3	−3 ≤ WHZ < − 2	−2 ≤ WHZ	Kappa (95% CI)
Whole group	(*n* = 1146)	(*n* = 1686)	(*n* = 7137)	0.408 (0.392–0.424)
MUAC < 115 (*n* = 1481)	47.2%	31.3%	21.5%	
	61.0%	27.5%	4.5%	
115 ≤ MUAC < 125 (*n* = 1685)	14.4%	34.0%	51.6%	
	21.1%	34.0%	12.2%	
125 ≤ MUAC (*n* = 6803)	3.0%	9.6%	87.4%	
	17.9%	38.6%	83.3%	
	WHZ < − 3	−3 ≤ WHZ < − 2	−2 ≤ WHZ	
Without edema	(*n* = 678)	(*n* = 1087)	(*n* = 5195)	0.398 (0.376–0.419)
MUAC < 115 (*n* = 760)	49.2%	28.7%	22.1%	
	55.2%	20.1%	3.2%	
115 ≤ MUAC < 125 (*n* = 1092)	14.0%	34.3%	51.6%	
	22.6%	34.5%	10.9%	
125 ≤ MUAC (*n* = 5108)	3.0%	9.7%	87.4%	
	22.3%	45.4%	85.9%	
	WHZ < − 3	−3 ≤ WHZ < − 2	−2 ≤ WHZ	
With edema	(*n* = 468)	(*n* = 599)	(*n* = 1942)	0.406 (0.379–0.433)
MUAC < 115 (*n* = 721)	45.1%	34.0%	20.9%	
	69.4%	40.9%	7.8%	
115 ≤ MUAC < 125 (*n* = 593)	15.0%	33.4%	51.6%	
	19.0%	33.1%	15.8%	
125 ≤ MUAC (*n* = 1695)	3.2%	9.2%	87.6%	
	11.5%	26.0%	76.5%	

First line (shaded in light grey): percentages in line; 2nd line: percentages in column; WHZ: Weight-for-height Z - score; MUAC: mid-upper arm circumference

We observed similar concordances by stratifying for sex, presence or absence of chronic malnutrition and age categories (Table Appendix 1).

### Concordance between MUAC and MUACZ for the diagnosis of acute malnutrition in children aged 6 to 59 months

As shown in Table 4, the concordance between MUAC and MUACZ was good except for subjects with edema for whom it was moderate. Additional analyzes showed that the concordance was moderate in children with chronic malnutrition, weak in the oldest children (24–59 months) (κ = 0.38) but excellent in younger children (κ = 0.80 for the 6–11 months and 0.86 among 12–23 months) (Table Appendix 2).

**Table 4:** Concordance between MUAC (mm) and MUACZ for the diagnosis of acute malnutrition in children aged 6 to 59 months admitted to the HPL from 1987 to 2008, eastern DRC

	MUACZ < − 3	−3 ≤ MUACZ < − 2	−2 ≤ MUACZ	Kappa (95% CI)
Whole group	(*n* = 2176)	(*n* = 2027)	(*n* = 5767)	0.604 (0.590–0.618)
MUAC < 115 (*n* = 1481)	93.9%	6.1%	0.0%	
	63.9%	4.5%	0.0%	
115 ≤ MUAC < 125(*n* = 1685)	40.5%	50.1%	9.4%	
	31.4%	41.6%	2.7%	
125 ≤ MUAC (*n* = 6803)	1.5%	16.1%	82.4%	
	4.7%	53.9%	97.3%	
	MUACZ < − 3	−3 ≤ MUACZ < − 2	−2 ≤ MUACZ	
Without edema	(*n* = 1059)	(*n* = 1311)	(*n* = 4590)	0.629 (0.611–0.647)
MUAC < 115 (*n* = 760)	89.6%	10.4%	0.0%	
	64.3%	6.0%	0.0%	
115 ≤ MUAC < 125(*n* = 1092)	30.2%	56.7%	13.1%	
	31.2%	47.2%	3.1%	
125 ≤ MUAC (*n* = 5108)	0.9%	12.0%	87.1%	
	4.5%	46.8%	96.9%	
	MUACZ < − 3	−3 ≤ MUACZ < − 2	−2 ≤ MUACZ	
With edema	(*n* = 1116)	(*n* = 716)	(*n* = 1177)	0.529(0.505–0.553)
MUAC < 115 (*n* = 721)	98.3%	1.7%	0.0%	
	63.5%	1.7%	0.0%	
115 ≤ MUAC < 125 (*n* = 593)	59.5%	37.9%	2.5%	
	31.6%	31.4%	1.3%	
125 ≤ MUAC (*n* = 1695)	3.2%	28.3%	68.6%	
	4.8%	66.9%	98.7%	

First line (shaded in light grey): percentages in line; 2nd line: percentages in column; MUAC: mid-upper arm circumference; MUACZ: mid-upper arm circumference for age Z score

### Concordance between WHZ and MUACZ for the diagnosis of acute malnutrition in children aged 6 to 59 months

We see in Table 5 that the WHZ and MUACZ had a low concordance, the lowest being in children with oedemas. This weak concordance was also observed in the oldest (24–59 months) and in those suffering from chronic malnutrition (respectively κ = 0.30 and κ = 0.33). However, among the youngest, it was moderate (κ = 0.43 for 6–11-month-olds and 0.42 for 12–23-month-olds) (Table Appendix 3).

**Table 5:** Concordance between WHZ and MUACZ for the diagnosis of acute malnutrition in children aged 6 to 59 months admitted to the HPL from 1987 to 2008, eastern DRC

	WHZ < − 3	−3 ≤ WHZ < − 2	−2 ≤ WHZ	Kappa (95% CI)
Whole group	(*n* = 1146)	(*n* = 1686)	(*n* = 7137)	0.363(0.347–0.379)
MUACZ < − 3 (*n* = 2175)	38.9%	32.9%	28.3%	
	73.7%	42.4%	8.6%	
−3 ≤ MUACZ < − 2 (*n* = 2027)	8.9%	27.3%	63.8%	
	15.8%	32.8%	18.1%	
−2 ≤ MUACZ (*n* = 5767)	2.1%	7.2%	90.7%	
	10.5%	24.8%	73.3%	
	WHZ < − 3	−3 ≤ WHZ < − 2	−2 ≤ WHZ	
Without edema	(*n* = 678)	(*n* = 1087)	(*n* = 5195)	0.391(0.371–0.411)
MUACZ < 115 (*n* = 1059)	42.8%	32.3%	24.9%	
	66.8%	31.5%	5.1%	
115 ≤ MUACZ < 125 (*n* = 1311)	9.6%	30.4%	60.0%	
	18.6%	36.6%	15.1%	
125 ≤ MUACZ (*n* = 4590)	2.2%	7.6%	90.3%	
	14.6%	31.9%	79.8%	
	WHZ < − 3	−3 ≤ WHZ < − 2	−2 ≤ WHZ	
With edema	(*n* = 468)	(*n* = 599)	(*n* = 1942)	0.288 (0.264–0.312)
MUACZ < 115 (*n* = 1116)	35.1%	33.4%	31.5%	
	83.8%	62.3%	18.1%	
115 ≤ MUACZ < 125 (*n* = 716)	7.7%	21.6%	70.7%	
	11.8%	25.9%	26.1%	
125 ≤ MUACZ (*n* = 1177)	1.8%	6.0%	92.2%	
	4.5%	11.9%	55.9%	

First line (shaded in light grey): percentages in line; 2nd line: percentages in column; WHZ: Weight-for-height Z - score; MUACZ: middle upper arm circumference for age Z -score

### Proportion of children with severe acute malnutrition by screening indicator in the group of children whose severe acute malnutrition was diagnosed by WHZ, MUAC and MUACZ taken in pairs

**Fig. 2:**
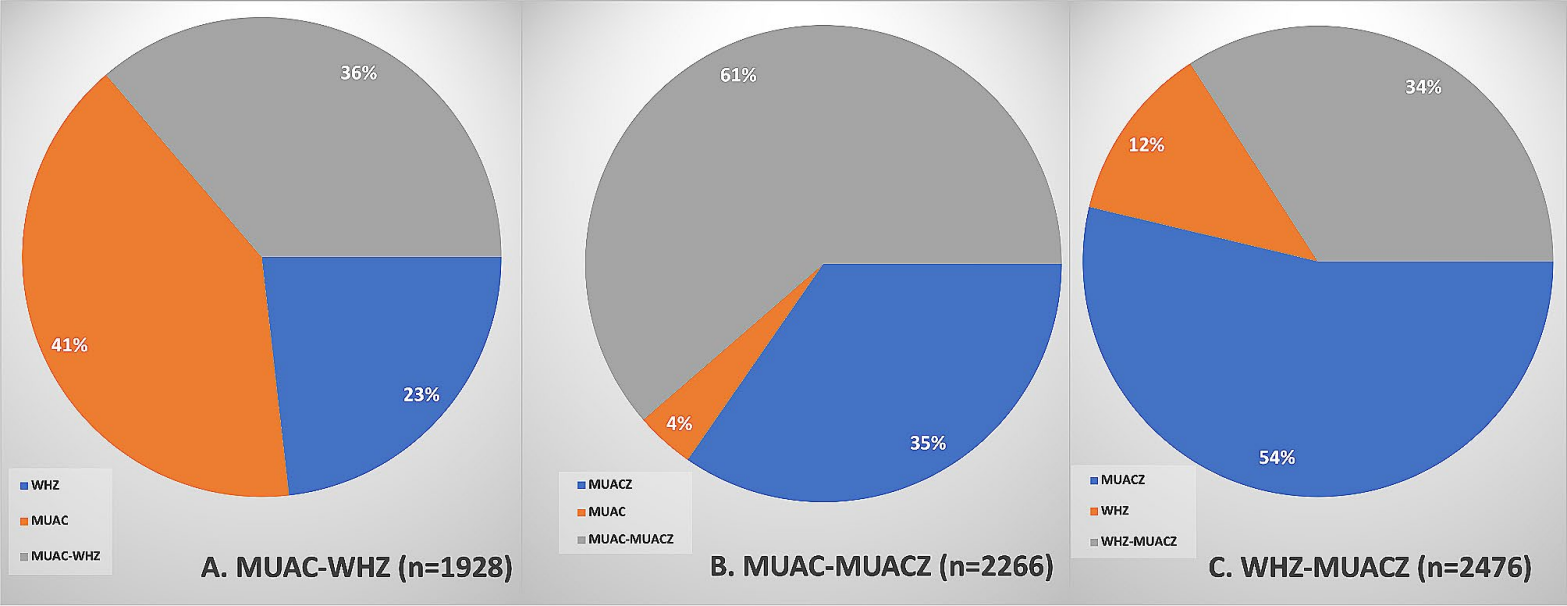
Proportion of children with severe acute malnutrition by screening indicator in the group of children whose severe acute malnutrition was diagnosed by WHZ, MUAC and MUACZ taken in pairs: **(A)** MUAC-WHZ, **(B)** MUAC-MUACZ and **(C)** WHZ- MUACZ, at HPL from 1987 to 2008, in South Kivu, eastern DR Congo

For the combination of WHZ and MUAC indicators, we found that 36% of children with SAM had both criteria at the same time. This proportion remained unchanged in the presence or absence of nutritional edema. Overall, MUAC alone was the indicator that recruited more SAM cases than WHZ, with 77% compared to 59% for WHZ alone (Fig. 2A). By stratifying by age, we observed the same trend among the youngest. However, among the oldest (24–59 months), the WHZ was the indicator that recruits more children than the MUAC, with 70% compared to 50% for the MUAC (Table Appendix 4).

For the combination of MUAC and MUACZ indicators, we found that 61% had both criteria at the same time. MUACZ alone was the indicator that recruited more SAM cases than MUAC, with 96% whereas MUAC alone recruited 65% (Fig. 2B). This difference was even more apparent in older children (MUACZ alone recruits 100% of SAM cases for children aged 24 to 59 months compared to 42% for MUAC alone). By stratifying, MUACZ recruited more SAM cases regardless of sex, presence or absence of nutritional edema, and chronic malnutrition. However, for younger children, MUAC was the indicator that recruits more SAM cases than MUACZ, with 96% compared to 82% for the MUACZ.

For the combination of WHZ and MUACZ indicators, we found that 34% of children fulfilled both criteria, regardless of the presence or absence of edema (Fig. 2C). Overall, MUACZ alone was the indicator that recruited more SAM cases, with 88% compared to 46% for WHZ alone. This was observed regardless of gender and age category. For children who had neither chronic malnutrition nor nutritional edema, the WHZ alone recruited slightly more children than the MUACZ with a difference of 5% (Table Appendix 5).

### Proportion of children with severe acute malnutrition by screening indicator in the group of children whose severe acute malnutrition was diagnosed by WHZ, MUAC and MUACZ consider at the same time

**Fig. 3:**
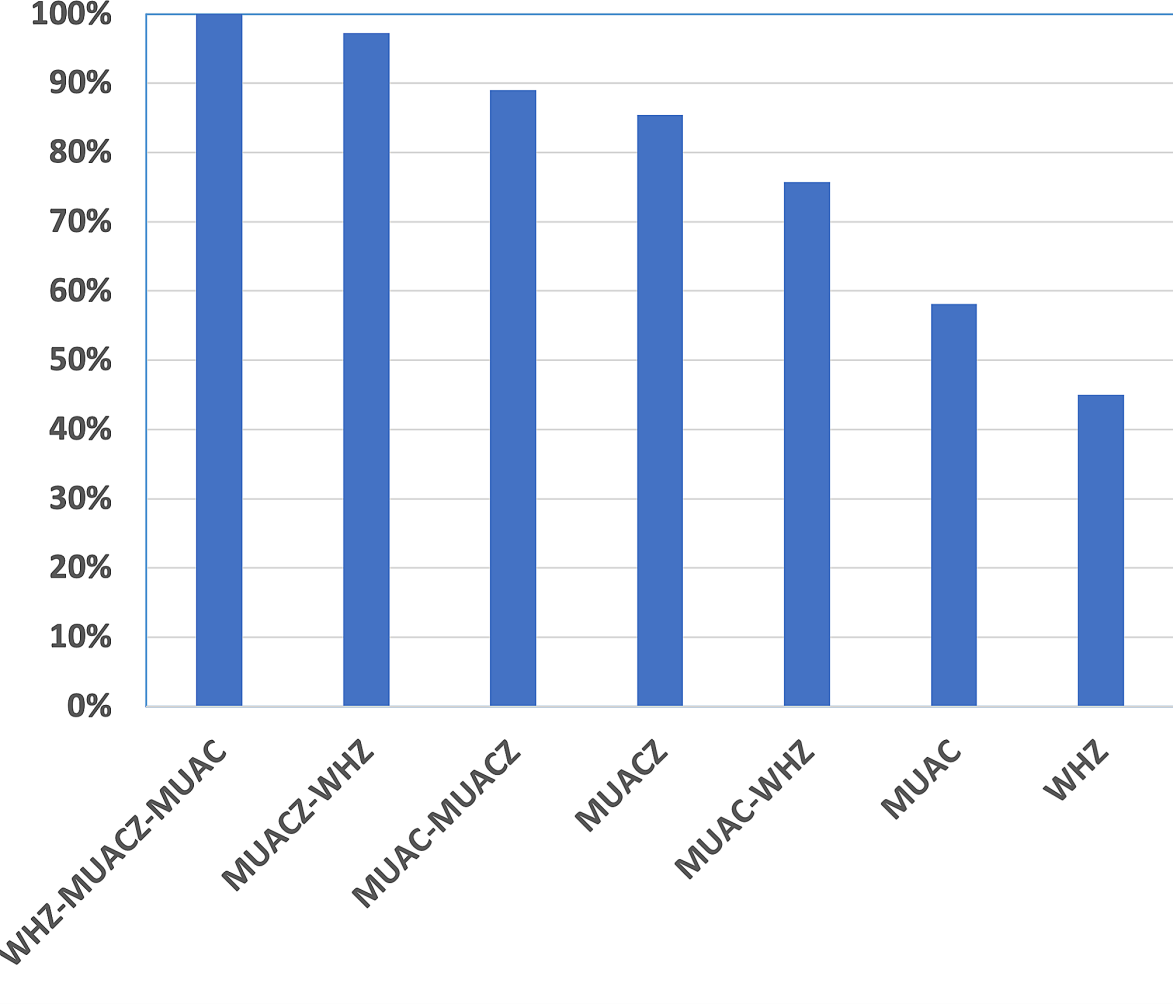
Proportion of children with severe acute malnutrition (*N* = 2547) by screening indicators and in different combinations in the group of children whose severe acute malnutrition was diagnosed by WHZ, MUAC and MUACZ at the same time, in South Kivu, eastern DR Congo

In the previous part, we used either the combination of MUAC alone with WHZ or MUACZ instead of MUAC alone. So, if we consider these three indicators at the same time (MUAC-MUACZ-WHZ), 25.5% of the population had at least one of these three criteria. In our cohort, MUACZ is the indicator that recruits the most SAM children (85%). By combining the indicators two by two, the MUACZ-WHZ group recruited more SAM cases (97%) with a difference of 22% compared with the classic MUAC-WHZ group (Fig. 3).

This difference was more pronounced in older children (100% of SAM detected with the MUACZ-WHZ combination against 59% with the MUAC-WHZ combination for children aged 23–59 months), children with nutritional edema (99% for the MUACZ-WHZ group with a difference of 27% with the MUAC-WHZ group) and those with chronic malnutrition (95% for the MUACZ-WHZ group with a difference of 31% with the MUAC-WHZ group). However, in younger children (6–11 months), MUAC alone became the indicator that recruited more SAM cases (85%) and, by combining the indicators, the MUAC-WHZ group recruited more SAM cases (97%) with only a 10% difference with the MUACZ-WHZ group (Table Appendix 6).

## Discussion

The objective of our study was to assess the concordance between the diagnostic indicators of SAM (WHZ and MUAC on the one hand, and WHZ and MUACZ on the other hand) in the endemic context of malnutrition in South Kivu where chronic malnutrition and the presence of nutritional edema predominate. The originality of this study consists of several points: to our knowledge, this study is the first in our region to have addressed concordance between WHZ and MUACZ for the diagnosis of SAM on a large database of children who have been followed for SAM in a tertiary level structure for twenty years.

### Screening for severe acute malnutrition in our cohort

When screening for severe acute malnutrition in our cohort, MUACZ was the indicator that recruited the most SAM cases followed by MUAC. Our results are consistent with those of Leidman et al., who found that MUACZ is the indicator that recruits more children with SAM in a study that analyzed 882 community-based surveys from 41 different countries [23]. However, our results contradict those of a study conducted in Somalia comparing estimates of acute malnutrition prevalence by each of the indicators for children aged 6 to 59 months, which found similar prevalence of SAM children for MUACZ (15.8%) and WHZ (16.1%) from a combined sample of 17 community-based surveys between 2007 and 2016, while the proportion of MUAC (7.8%) was much lower [26].

Regarding the diagnosis of SAM, we have illustrated above that many studies have already shown that both WHZ and MUAC criteria do not recruit exactly the same children with SAM. By combining WHZ and MUAC for the diagnosis of SAM, we found that 36% of children met both criteria regardless of the presence or absence of edema. Our results are similar to most other studies, which have consistently shown a proportion of overlap below 40%, although varying by country (22–23). Some studies have shown lower proportions than ours, notably the study by Grellety et al. [19], where only 16.5% of children defined as having SAM were diagnosed with both low MUAC and low WHZ. Similarly, Roberfroid et al. [21] found that only 28.5% of children defined as suffering from global acute malnutrition met the criteria for both at the same time. In this latter study, the proportion of SAM is not known but is expected to be lower than that of GAM. Our study further demonstrated that MUAC alone recruited more children with SAM than WHZ alone, a finding consistent with previous studies [14, 19].

### Concordance between screening criteria for severe acute malnutrition in our cohort

The consistency between different methods is a crucial requirement for ensuring the quality of diagnostic methods [46]. Examining how well the methods agree involves measuring how well the results of different test methods agree, not just the association or correlation of the results. To do this, we used Cohen’s kappa coefficient (κ); which remains the most commonly used concordance measure (46–47). It represents the chance-adjusted proportional agreement for the categorical variables (48–45). As kappa is affected by prevalence and predictive values, it is not appropriate to compare kappa measurements between different studies [48, 49]. However, it can provide more information than a simple calculation of the crude proportion [49].

### Concordance between WHZ and MUAC

In our study, we found that the WHZ and MUAC showed a moderate concordance (0.21 ≤ κ ≤ 0.40) and that this concordance was lower in girls and in older children (24–59 months). This concordance was moderate for young children. That has also been reported in other studies [16–23]. As MUAC increases with age [50, 51], a single MUAC threshold value is likely to categorize more young children as wasted than older ones. However, the MUAC is simple to use although there is a possibility of making measurement errors with the use of bracelets. The WHZ on the other hand is not easy to use because it requires not only accurate measurements of weight and height but also reading with interpretation of growth curves [52, 53]. It has the advantage of evaluating nutritional status in relation to WHO growth standards and does not need to know the age for its interpretation. Additionally, the WHZ is largely influenced by leg length and may also decrease its ability to identify high-risk children [51]. The weight and/or height of children with edema may be overestimated because children with edema may appear slightly taller than those without edema due to standing on their swollen limbs. This may also decrease the WHZ’s ability to identify children with AM in an area with high prevalence of Kwashiorkor. Another explanation would be the predominance of chronic malnutrition. We have shown that in South Kivu, approximately 1 child out of 2 suffers from CM [37]. Children with chronic malnutrition have a shorter height compared to the expected height for their age. However, in cases of severe acute malnutrition, which is sometimes associated with inadequate weight for height, children with a short stature due to chronic malnutrition tend to have a weight that is in proportion to their height if they experience weight loss. Consequently, the calculation of weight-for-height Z-score (WHZ) may be influenced by chronic malnutrition. This could also be obvious because MUAC attempts to summarize fat and muscle wasting while weight for height attempts to summarize soft tissue versus hard tissue deficit.

### Concordance between WHZ and MUACZ

Other researchers have justified this discrepancy on the grounds that MUAC does not take age into account like the WHZ and have suggested that MUACZ may have greater diagnostic concordance with WHZ than MUAC due to the fact that MUACZ is adjusted for age and sex [23, 25–27]. However, the calculation of the MUACZ indicator is similar to the WHZ, both of which compare a child’s anthropometric measurements to an international reference population to classify acute malnutrition in children aged 6–59 months. On the other hand, we found that the concordance between WHZ and MUACZ was poor overall, but moderate in children without edema and in children aged between 6 and 23 months. Compared to the different proportions, our data found that by combining MUACZ and WHZ for the diagnosis of SAM, only 34% of children had both criteria at the same time, but this proportion was 35% in subjects without edema, of almost similar proportions to those found by both the WHZ and MUAC. Our results differ from those found in Somalia by Custodio et al. [26], who found that the proportion of acutely malnourished children identified by WHZ and MUACZ (28.3%) was higher than the proportion of children identified by WHZ and MUAC (18.1%).

MUACZ alone is the indicator that recruits more children with SAM, taking into account or not the presence of edema, than WHZ alone and MUAC alone. We also note that the children recruited by WHZ and MUACZ at the same time are more numerous than those recruited with WHZ alone, MUAC alone, or even by MUAC and WHZ at the same time. This is observed regardless of sex, age, chronic malnutrition, and the presence or absence of nutritional edema.

### Concordance between MUAC and MUACZ

Since the development and publication of the MUACZ benchmark data in 1997 [54], little research has explored the association between MUAC and MUACZ. In Nigeria, a study calculated the diagnostic concordance of these two indicators and found that 35.3% of SAM children aged 6–59 months were diagnosed with both MUACZ and MUAC; 17.7% were MUACZ only and 47.0% were MUAC only [55]. Another study found that overall MUACZ identified more children than MUAC and that the proportion of children with SAM diagnosed by MUACZ alone in almost all surveys was significantly higher than MUAC alone; this finding was very consistent across regions [23]. Therefore, it may be possible to devise a relatively reliable formula for converting MUAC to MUACZ, as was done previously to convert National Center for Health Statistics (NCHS) growth reference estimates to estimates using WHO growth standards, where a high degree of fit was observed [56].

Our study showed that MUACZ identifies more cases of severe acute malnutrition (SAM) than MUAC alone, and the overlapping proportion between the two indicators for SAM was 61.34%. By measuring concordance, our study also reveals a moderate concordance between MUAC and MUACZ. This concordance was good for children who do not have nutritional edema except for those aged 24–59 months and those with chronic malnutrition. It becomes excellent for children without edema and aged between 12 and 23 months. Our study showed that there is an association between MUACZ and MUAC although it is MUACZ that recruits more children with SAM. This association is influenced by age. The concordance between these two criteria was greater in subjects under 24 months. Studies evaluating the capacity of these two diagnostic criteria to predict the cases of death of children with SAM, the prognosis in the short, medium, and long term, are to be encouraged in order to properly decide on the usefulness of using one over the other because the main objective of SAM management programs is to prevent the mortality of children with SAM. It should be noted that the use of MUAC is very easy in the field although there may still be some minor measurement errors, not requiring much effort like MUACZ and WHZ to read the growth curve but its use alone could miss a significant number of children with SAM who could be identified by the other criteria (MUACZ and/or WHZ) and who could have a worse prognosis in hospitalization. The current national protocols in the DRC explicitly advocate for the simultaneous use of both WHZ and MUAC in the admission process for severe acute malnutrition treatment. This complementary approach allows for a more comprehensive identification of children in need of intervention, and we advocate for maintaining this recommendation to ensure adequate care in our local context. However, it is important to note that despite these national criteria (using MUAC and WHZ), specific programs in our region, such as emergency interventions and simplified programs supported by international non-governmental organizations, predominantly encourage the exclusive use of MUAC as an admission indicator. We wish to express our reservation in this regard, as not only do MUACZ and WHZ show weak concordance, but our previous study on this cohort [40] has also demonstrated that WHZ is more strongly associated with mortality than MUAC alone. Our data clearly showed that despite the ease of use of MUAC alone, many children with SAM will not be enrolled in care programs because of the exclusive use of MUAC. Similarly, the use of MUAC and WHZ also does not identify all children with SAM since in some regions like ours, MUACZ is the indicator that recruits more wasted children. This shows that age adjustment is very important in a region with a high prevalence of kwashiorkor and chronic malnutrition. In the context of assessing malnutrition, MUACZ could be used as a simple and quick anthropometric indicator. It provides an estimate of muscle mass and lean body mass. MUACZ is particularly useful in situations where oedema, such as that seen in kwashiorkor, may distort the assessment of body weight. Similarly for chronically malnourished children who have a short stature, the MUACZ would be an ideal tool because short stature due to chronic malnutrition can also bias the WHZ assessment. Thus, MUACZ becomes a valuable tool for assessing severe acute malnutrition outside the Kwashiorkor. MUACZ assesses whether a child’s arm circumference is less or greater than expected for age and sex, which can improve the use of fixed MUAC thresholds for all children aged 6 to 59 months. This will help health professionals identify children in need of appropriate nutritional interventions tailored to their specific condition.

### Practical considerations

Our findings underscore the crucial importance of maintaining the simultaneous use of both MUAC and WHZ criteria in the management protocols, ensuring comprehensive inclusion of all children in malnutrition intervention programs, especially in our context where kwashiorkor and chronic malnutrition prevail. In response to this reality, our study advocates for the systematic implementation of MUACZ, standing out as an exceptionally effective tool for recruiting a larger number of children into acute malnutrition intervention programs, offering a pragmatic approach tailored to our local specificities. In community-based programs, our results highlight that MUACZ outperforms MUAC alone in terms of diagnostic performance. However, for maximal optimization of MUAC-only programs, it is recommended to consider integrating complementary criteria such as WHZ or MUACZ. This proposition is grounded in the recognition that while MUAC-only programs with a simplified approach demonstrate efficacy, continuous improvement is possible by enabling better identification of all children suffering from malnutrition, including those who may not be diagnosed by MUAC alone. To ensure the success of these recommendations in the field, it is imperative to consider the practical aspects of implementation. This includes adequate training for health personnel and community workers, ensuring the availability of appropriate measuring equipment, and addressing operational constraints specific to each context. By incorporating these elements, we anticipate a significant enhancement in the quality and coverage of malnutrition intervention programs, thereby more effectively meeting the needs of children in our community.

### Study limitations

Some limitations are worth mentioning. Information on the shape and length of each child’s legs is not known. Some authors suggest that the shape and length of children’s legs would influence WHZ’s ability to recruit MAG children. Despite these limitations, our study remains one of the few to address this topic on a cohort of children followed in a tertiary level structure with such a large sample. In addition, some records were excluded at the start of the study because subjects had incomplete data, left during hospitalization, or were transferred to another facility, we do not know how the data on these subjects would have influenced our results, but as the number is small, we do expect a high impact on the results. Finally, because our study uses hospitalized data, hospitalized children are often those with more serious health problems or complications related to severe acute malnutrition. Therefore, data collected in a hospital setting may not be representative of the entire population of malnourished children.

## Conclusion

We found that the concordances between MUAC and WHZ on the one hand and between MUACZ and WHZ on the other hand were weak in our region. Concordance between WHZ and MUACZ improved slightly in young subjects and in those without nutritional edema. MUACZ was the indicator that recruited more wasted children than the other two indicators in our region with a high prevalence of kwashiorkor and chronic malnutrition. MUAC and MUACZ had a moderate concordance, reaching its highest values in young subjects under 24 months. In the field, the MUACZ will not be easy to use like the WHZ since it requires adjustment for age and the use of reference curves. But in the therapeutic intensive care units (ICU) of malnutrition this remains essential. It is strongly recommended to promote suggestions to simplify the use of MUACZ, especially in areas with a high prevalence of edema. It should also be noted that there is also a need for further research assessing the mortality risk of MUACZ, as has already been documented for MUAC and WHZ. We hope that with the evolution of informatics, simple means will be available to facilitate the use of all diagnostic indicators of SAM in the community, but this cannot prevent the use of MUACZ in the community-based acute malnutrition management program.

### Electronic supplementary material

Below is the link to the electronic supplementary material.

Supplementary Material 1

## Data Availability

All data generated or analyzed during this study are included in this published article.

## Abbreviations

DHS: Demographic survey
HPL: Hôpital Pédiatrique de Lwiro
SAM: Severe Acute Malnutrition
MAM: Moderate Acute Malnutrition
MAG: Global Acute Malnutrition
WHO: World Health Organization
PCMA: community management of Acute Malnutrition
DRC: Democratic Republic of Congo
WHZ: Weight-for-height Z -score
MUAC: middle upper arm circumference
MUACZ: middle upper arm circumference for age Z score
k: kappa coefficient
CEMUBAC: Centre Scientifique et Médical de l’Université libre de Bruxelles pour ses Activités de Coopération
HZ: Health Zone
AM: Acute malnutrition
CM: Chronic malnutrition
WAZ: Weight for age Z-score
HAZ: height for age Z-score
CRSN-Lwiro: Centre de Recherche en Sciences Naturelles Lwiro
NCHS: National Center for Health Statistics
