# Supplementary material for: Arm circumference for age, arm circumference and weight-for-height z-score for the evaluation of severe acute malnutrition: a retrospective cohort study in eastern Democratic Republic of Congo
Source: BMC Public Health. 2024 Feb 23;24:587. doi: 10.1186/s12889-024-18083-y (PMC10885520; doi:10.1186/s12889-024-18083-y)
Supplement: Supplementary file 1 — Supplementary Material 1 [file 12889_2024_18083_MOESM1_ESM.docx]

**Table Appendix 1.** Concordance between WHZ and MUAC for the diagnosis of Acute Malnutrition in children aged 6 to 59 months by stratifying for sex, presence or absence of chronic malnutrition and age categories: coefficients kappa.

|  | Entire group | Without edema | With edema |
| --- | --- | --- | --- |
| Sex |  |  |  |
| Boy | 0.428 | 0.409 | 0.448 |
| Girl | 0.388 | 0.388 | 0.364 |
| Age (months) |  |  |  |
| 6-11 | 0.403 | 0.400 | 0.339 |
| 12-23 | 0.448 | 0.437 | 0.416 |
| 24-59 | 0.381 | 0.363 | 0.389 |
| CM |  |  |  |
| yes | 0.399 | 0.386 | 0.411 |
| No | 0.379 | 0.375 | 0.350 |

*CM=Chronic malnutrition

**Table Appendix 2.** Concordance between MUAC and MUACZ for the diagnosis of Acute Malnutrition in children aged 6 to 59 months by stratifying for sex, presence or absence of chronic malnutrition and age categories: coefficients kappa.

|  | Entire group | Without edema | With edema |
| --- | --- | --- | --- |
| Sex |  |  |  |
| Boy | 0.581 | 0.627 | 0.474 |
| Girl | 0.628 | 0.632 | 0.586 |
| Age (months) |  |  |  |
| 6-11 | 0.799 | 0.789 | 0.834 |
| 12-23 | 0.861 | 0.858 | 0.842 |
| 24-59 | 0.375 | 0.348 | 0.370 |
| CM |  |  |  |
| yes | 0.565 | 0.595 | 0.509 |
| No | 0.688 | 0.676 | 0.687 |

*CM=Chronic malnutrition

**Table Appendix 3**. Concordance between WHZ and MUACZ for the diagnosis of Acute Malnutrition in children aged 6 to 59 months by stratifying for sex, presence or absence of chronic malnutrition and age categories: coefficients kappa.

|  | Entire group | Without edema | With edema |
| --- | --- | --- | --- |
| Sex |  |  |  |
| Boy | 0.370 | 0.391 | 0.307 |
| Girl | 0.356 | 0.390 | 0.267 |
| Age (months) |  |  |  |
| 6-11 | 0.432 | 0.430 | 0.359 |
| 12-23 | 0.429 | 0.429 | 0.372 |
| 24-59 | 0.300 | 0.340 | 0.245 |
| CM |  |  |  |
| yes | 0.332 | 0.366 | 0.275 |
| No | 0.394 | 0.385 | 0.380 |

*CM=Chronic malnutrition

**Table Appendix 4. Combination of WHZ and MUAC criteria for the diagnosis of SAM in all children admitted on the basis of the presence or absence of oedema by sex, age category and chronic malnutrition at HPL from 1987 to 2008**

|  |  |  | With edema | | |  |  |  |
| --- | --- | --- | --- | --- | --- | --- | --- | --- |
|  |  | Sex | | Age category (Months) | | | CM | |
| WHZ-MUAC | all | Girl | Boy | 6-11 | 12-23 | 24-59 | CM | wihout CM |
| n | 1928 | 961 | 967 | 486 | 651 | 791 | 1629 | 299 |
| % of WHZ only | 23 | 16 | 31 | 13 | 17 | 34 | 21 | 35 |
| % of MUAC only | 41 | 47 | 33 | 51 | 41 | 34 | 41 | 40 |
| % of WHZ and MUAC | 36 | 37 | 36 | 36 | 42 | 32 | 38 | 25 |
|  |  |  | Without edema | | |  |  |  |
| WHZ-MUAC | 1064 | 519 | 545 | 394 | 334 | 336 | 845 | 219 |
| % of WHZ only | 29 | 20 | 37 | 15 | 24 | 50 | 26 | 40 |
| % of MUAC only | 36 | 43 | 30 | 50 | 36 | 20 | 36 | 36 |
| % of WHZ and MUAC | 35 | 37 | 33 | 35 | 40 | 30 | 38 | 24 |

WHZ: Weight-for-height Z - score; MUACZ: middle upper arm circumference for; CM: chronic malnutrition

**Table Appendix 5. Combination of WHZ and MUACZ criteria for the diagnosis of SAM in all children admitted on the basis of the presence or absence of oedema by sex, age category and chronic malnutrition at HPL from 1987 to 2008**

|  |  |  | With edema | |  |  |  |  |
| --- | --- | --- | --- | --- | --- | --- | --- | --- |
|  |  | Sex | | Age category (Month) | | | CM |  |
| MUACZ-WHZ | all | Girl | Boy | 6-11 | 12-23 | 24-59 | CM | wihout CM |
| n | 2475 | 1149 | 1326 | 438 | 687 | 1350 | 2169 | 306 |
| % of WHZ only | 12 | 11 | 13 | 17 | 15 | 10 | 10 | 32 |
| % of MUACZ only | 54 | 56 | 52 | 45 | 44 | 61 | 55 | 42 |
| % of WHZ and MUACZ | 34 | 33 | 35 | 38 | 41 | 29 | 35 | 26 |
|  |  |  | Without edema | |  |  |  |  |
| MUACZ-WHZ | all | Girl | Boy | 6-11 | 12-23 | 24-59 | CM | wihout CM |
| n | 1284 | 568 | 716 | 350 | 351 | 583 | 1070 | 214 |
| % of WHZ only | 18 | 17 | 18 | 20 | 21 | 14 | 13 | 40 |
| % of MUACZ only | 47 | 47 | 47 | 44 | 39 | 54 | 50 | 35 |
| % of WHZ and MUACZ | 35 | 36 | 35 | 36 | 40 | 32 | 37 | 25 |

WHZ: Weight-for-height Z - score; MUACZ: middle upper arm circumference for age Z -score; CM: chronic malnutrition

**Table Appendix 6. Combination of WHZ, MUAC and MUACZ criteria for the diagnosis of SAM in all children admitted on the basis of the presence or absence of oedema by sex, age category and chronic malnutrition at HPL from 1987 to 2008**

| WHZ-MUACZ-MUAC |  | Edema | | **CM** | | **Age (Months)** | | |
| --- | --- | --- | --- | --- | --- | --- | --- | --- |
|  | All | Yes | No | Yes | No | 6-11 | 12-23 | 24-59 |
| MUAC | 58% | 60% | 57% | 58% | 59% | 85% | 77% | 38% |
| MUAC-MUACZ | 89% | 94% | 85% | 91% | 74% | 88% | 87% | 91% |
| MUAC-WHZ | 76% | 72% | 79% | 67% | 91% | 97% | 93% | 59% |
| MUACZ | 85% | 93% | 79% | 89% | 63% | 73% | 85% | 91% |
| MUACZ-WHZ | 97% | 99% | 95% | 98% | 93% | 88% | 99% | 100% |
| WHZ | 45% | 39% | 50% | 44% | 54% | 48% | 55% | 39% |
| WHZ-MUACZ-MUAC | 100% | 100% | 100% | 100% | 100% | 100% | 100% | 100% |

WHZ: Weight-for-height Z - score; MUACZ: middle upper arm circumference for age Z -score; CM: chronic malnutrition; MUACZ: middle upper arm circumference.
